# Investigating the Latent Structure of Executive Function in the Delis–Kaplan Executive Function System Using Cattell–Horn–Carroll Theory

**DOI:** 10.1177/10731911231161779

**Published:** 2023-04-03

**Authors:** Rachel T. Furey, Stephen C. Bowden, Paul A. Jewsbury, Navaneetham J. Sudarshan, Madeleine L. Connolly

**Affiliations:** 1 The University of Melbourne, Australia; 2 St. Vincent’s Hospital Melbourne, Australia

**Keywords:** factor analysis, executive function, D-KEFS, neuropsychological testing, Cattell–Horn–Carroll

## Abstract

**Objective:**

To replicate a seven-factor model previously reported for the Delis–Kaplan Executive Function System (D-KEFS).

**Method:**

This study used the D-KEFS standardization sample including 1,750 non-clinical participants. Several seven-factor models previously reported for the D-KEFS were re-evaluated using confirmatory factor analysis (CFA). Previously published bi-factor models were also tested. These models were compared with a three-factor a priori model based on Cattell–Horn–Carroll (CHC) theory. Measurement invariance was examined across three age cohorts.

**Results:**

All previously reported models failed to converge when tested with CFA. None of the bi-factor models converged after large numbers of iterations, suggesting that bi-factor models are ill-suited to represent the D-KEFS scores as reported in the test manual. Although poor fit was initially observed for the three-factor CHC model, inspection of modification indices showed potential for improvement by including method effects via correlated residuals for scores derived from similar tests. The final CHC model showed good to excellent fit and strong metric measurement invariance across the three age cohorts with minor exceptions for a subset of Fluency parameters.

**Conclusions:**

CHC theory extends to the D-KEFS, supporting findings from previous studies that executive functions can be integrated into CHC theory.

The Delis–Kaplan Executive Function System (D-KEFS; Delis et al., 2001) is a popular neuropsychological battery used to screen for executive impairment in patients with brain disorders. The D-KEFS includes nine tests that can be administered individually, or as a selection of tests at the discretion of the clinician. According to the test authors, D-KEFS scores are not intended to represent specific executive function constructs, but are presumed to be influenced by a range of executive processes, with no option for composite scoring provided in the test manual (Delis et al., 2001). Rather, according to the test authors, the D-KEFS should be used as a broad preliminary measure in clinical hypothesis formation and subsequently followed by more targeted neuropsychological tests (Delis et al., 2004).

The D-KEFS has been criticized for a lack of underlying measurement model, which makes the scores ambiguous and difficult to interpret in relation to established cognitive ability theory (R. G. Floyd et al., 2010; Jewsbury et al., 2016, 2017). The best-known and empirically supported model of executive function proposes that there are three executive function constructs, commonly termed switching, inhibiting, and updating (Miyake et al., 2000). Data from multiple studies suggest that these executive function constructs can be integrated into the broader Cattell–Horn–Carroll (CHC) model of cognitive abilities (Jewsbury et al., 2016). The CHC model is supported by a wealth of factor analytic evidence and is increasingly used as a theoretical basis for cognitive test interpretation in clinical settings (Schneider & McGrew, 2018). The CHC abilities most relevant to executive function constructs are fluid reasoning (Gf), which describes the ability to solve novel problems using deliberate and controlled procedures such as induction and deduction, processing speed (Gs), which is the ability to control attention to perform simple cognitive tasks quickly, and retrieval fluency (Gr), which describes the speed and fluency with which an individual can retrieve information. In a previous factor analysis comparing the switching, inhibiting, and updating model to CHC theory, Jewsbury et al. (2016) suggested that updating can be integrated with working memory (Gwm or Gsm), inhibiting can be integrated with processing speed (Gs) as a broad ability, and switching is a narrow factor within processing speed (Gs).

Executive function is commonly assessed alongside other cognitive abilities in neuropsychological assessments, so it is desirable to have a general measurement model that can be used as a coherent theoretical basis for all interpretations of test performance. As typically defined, a measurement model demonstrates the relationship between test scores and the underlying constructs, and is usually evaluated using confirmatory factor analysis (CFA; Widaman & Reise, 1997). There is some evidence to suggest that a three-factor model consistent with the switching, inhibiting, and updating model may fit the D-KEFS scores (Karr et al., 2019; Latzman & Markon, 2010) in which case the D-KEFS scores may be integrated with the CHC model. However, methodological limitations regarding the use of factor analysis in previous studies reduce the extent to which published models may be useful for clinical interpretations.

Latzman and Markon (2010) used exploratory factor analysis (EFA) and determined the number of factors to extract based on eigenvalues and Velicer’s minimum average potential criterion (Velicer et al., 2000) rather than the recommended approach of using global fit statistics produced by the maximum likelihood estimator (J. D. Brown, 2009; Costello & Osborne, 2005; F. J. Floyd & Widaman, 1995; Henson & Roberts, 2006). As a consequence, Latzman and Markon (2010) reported different EFA solutions in each of the three age groups they analyzed, being the age groups reported in the D-KEFS manual.

Karr et al. (2019) found support for a three-factor, bi-factor model for the D-KEFS with primary factors showing conceptual consistency with switching and inhibiting executive function constructs. The third factor in this model was conceptualized as fluency, as the D-KEFS includes tasks that measure fluency but not updating. Because fluency and updating have some conceptual overlap, the authors used fluency as proxy for updating. However, previous work with multiple independent data sets suggests that fluency should not be used as a proxy for updating because the latter is best interpreted as synonymous with working memory, and fluency is a CHC distinct construct (Jewsbury et al., 2016). In addition, a bi-factor model may not be ideal for clinical use, as bi-factor models do not provide a clear basis for representing executive function theory or assessment practice. According to bi-factor models, the reliable variance in any test score can be interpreted as representing two uncorrelated abilities, in this context the general executive ability or the test-specific executive ability. The derivation of two ability measures from one test score may create ambiguity for clinical interpretation. There is also evidence to suggest that bi-factor models may be artificially well-fitting. Simulation studies have shown that comparative fit indices favor bi-factor models when sample sizes are small, or when model misspecification is present, even when the data are generated from a known correlated factors model (Greene et al., 2019). Despite being the final preferred model, Karr et al. (2019) found the bi-factor model failed to converge in 65% of bootstrapping samples, which may indicate the model is not well specified (appropriate) for the data. It is worth noting that although Karr et al. (2019) used the D-KEFS standardization sample, the model was based on data from the 20- to 49-year cohort using a subset of the 16 main achievement scores as dependent variables. Karr and colleagues also used transformed (residualized) scores, not the scores used by clinicians in practice. Therefore, the relevance of their model to clinical practice, statistical identification issues aside, is not clear.

Other factor analytic studies have sought more complex factor solutions for the D-KEFS in support of a cognitive processing theory potentially influenced by numerous executive processes. A notable example is McFarland (2020), who sought to demonstrate the hypothesized complexity of the D-KEFS by using a number of exploratory methods to model the 16 D-KEFS scores. McFarland specifically criticized the approach of Jewsbury et al. (2016), arguing that executive functions should be considered distinct from CHC cognitive ability constructs and that a complex factor structure should be sought rather than a simpler structure. This assumption was based on the premise that each D-KEFS score is influenced by a complex mixture of several executive abilities, as argued by the D-KEFS authors (Delis et al., 2001). Using the D-KEFS standardization sample data, McFarland proposed that a seven-factor model showed the best fit and provided three different seven-factor models using ad hoc and post hoc model selection criteria, termed “forwards,” “backwards,” and “backwards-correlated.” However, McFarland’s approach to factor analysis was suboptimal. Researchers conducting factor analysis are required to make multiple decisions regarding technique, with a number of options available at each step (Watkins, 2018). Different techniques can lead to different conclusions, so it is essential that researchers make these decisions carefully and follow established guidelines to maximize transparency and replicability (e.g., T. A. Brown, 2015; Kline, 2015; Widaman, 2012). Some of McFarland’s analysis decisions do not follow recommend guidelines, and many analytic steps lacked adequate description or justification. For example, McFarland initially used principal component analysis, which is not strictly regarded as factor analysis (Floyd & Widaman, 1995), used obsolete model selection criteria such as eigenvalues, and imposed post hoc modifications, specific details of which were not clear from the way the results were reported. The resulting models were difficult to interpret, involving multiple loadings on multiple factors, and thus may not be statistically identified or replicable. An identified model in CFA is a model with sufficient indicators (or test scores) to uniquely estimate the parameters of the model.

Nevertheless, McFarland (2020) stated that Jewsbury and colleagues (2016) proposed integration of executive function tests with CHC theory reflected an over-reliance on factor analysis, “factor analysis by itself may be misleading as far as theory development is concerned” (McFarland, 2020, pp. 9–10). However, McFarland’s argument does not appear to appreciate that factor analysis is complementary to criterion-related validity, and careful factor analysis reflects the best available implementation of the convergent and discriminant validity framework that was advocated by MacCorquodale and Meehl (1948) to refine theoretical understanding in psychology (Strauss & Smith, 2009). In these terms, McFarland created a “straw-person” argument when criticizing the view that factor analysis is both necessary and sufficient to demonstrate construct validity.

To date, a latent variable model consistent with established theory and derived from the recommended factor analysis approach has not been reported for the D-KEFS despite the popularity of the battery. The American Psychological Association guidelines for psychological assessment recommend that test selection for clinical use be based on demonstrated evidence of construct validity and that test scores be interpreted and reported with reference to meaningful evidence and theory, in line with established guidelines for construct validity (American Psychological Association, APA Task Force on Psychological Assessment and Evaluation Guidelines, 2020; Strauss & Smith, 2009). Therefore, there is a need to examine the quality of the factor analytic evidence available for the D-KEFS.

The aim of this study was to use CFA to investigate the replicability of the seven-factor model for the D-KEFS suggested by McFarland (2020) and to compare that model with a three-factor model based on CHC abilities (Jewsbury et al., 2016). A switching, inhibiting, and updating model was not tested in the current study because the D-KEFS does not include tests that clearly correspond to all three constructs. Specifically, there is no coverage of the updating or working memory construct (Miyake et al., 2000). Although, as noted, fluency was used as a proxy for updating by Karr et al. (2019), differences in methodological approach limit the comparability of this study to ours, and a full replication of Karr et al. (2019) was not desirable, on conceptual grounds, because of the ambiguity in definition of updating used by Karr et al. In addition, Karr et al. used adjusted raw scores, scores to which we do not have access. However, to be comprehensive in evaluation of previously published models, bi-factor model variants of our three-factor CHC models were evaluated. In addition, we sought to test a direct replication of the preferred model of Karr et al. using the scores employed by clinicians to interpret D-KEFS performance.

To test the generality of a best-fitting model, we also undertook measurement invariance analysis across the three age cohorts in the D-KEFS standardization sample. Analysis of measurement invariance allows evaluation of the hypothesis that the relationship between test scores and the respective factor scores is numerically equivalent across different populations or cohorts and, when established, permits generalization of construct validity research and uncomplicated comparison of means for the identification of deficit or other population or cohort effects (Widaman & Reise, 1997). Available research suggests that measurement invariance of cognitive abilities is the rule across diverse clinical and national cohorts, but needs to be established before construct validity generalization can be assumed (AERA et al., 2014; Jewsbury et al., 2016, 2017).

## Method

### Participants and Data Analysis

In this section, we report how we determined our sample size, all data exclusions, all manipulations, and all measures in the study. This study used the D-KEFS standardization sample correlation matrices reported in the technical manual (Delis et al., 2001), the same data that were used by McFarland (2020). The D-KEFS standardization sample includes 1,750 non-clinical participants who were representative of the national population in the United States at the time of recruitment. The sample data is divided into three age cohorts, with the sample size for each age cohort reported here as a range because correlations in each cohort were based on different sample sizes. The full sample includes 8- to 19-year-olds, (*N* = 703–875), 20- to 49-year-olds (*N* = 361–425), and 50- to 89-year-olds (*N* = 326–450).

All analyses were completed using Mplus Version 8 using maximum likelihood estimation (Muthén & Muthén, 2017). CFA models were derived from covariance matrices using the means, standard deviations, and correlation matrices for each of the 16 main achievement scores reported in the test manual (Tables 3.28–3.30; Delis et al., 2001). In line with the recommendations of Marsh (1998), we specified the number of observations based on the smallest *N* reported for each age cohort for all model estimates (8- to 19-year-olds, *n* = 702; 20- to 49-year-olds, *n* = 361; 50- to 89-year-olds, *n* = 326). The Proverbs test score was excluded from the 8- to 19-year cohort analysis due to a small size (*N* = 165) because this test is described as only suitable for individuals aged 16 and over (Delis et al., 2001).

The primary objective of this study was to replicate the seven-factor models reported by McFarland (2020) as “best-fitting” using CFA and compare the resulting models to a three-factor model specified a priori and based on CHC theory (Jewsbury et al., 2016). Since the recommended D-KEFS scoring produces multiple scores from some tests, it was anticipated that correlated residuals indicative of method effects would be observed (Cole et al., 2007; Millsap, 1995). Therefore, it was anticipated that inclusion of correlated residual would improve model fit, and it was further anticipated that correlated residuals that replicated across age-group samples would be incorporated into the best-fitting CFA model. All CFA models were evaluated with the root mean square error of approximation (RMSEA), the comparative fit index (CFI), and the Tucker–Lewis Index (TLI). Goodness of fit was defined according to Hu and Bentler’s (1999) guidelines, with good fit indicated by RMSEA values below 0.06, and CFI and TLI values above 0.95. Acceptable fit was defined as RMSEA values below 0.08, and CFI and TLI values above 0.90.

### Measures

The D-KEFS includes nine individual tests from which 16 main achievement scores can be derived. The nine tests are Sorting, Trail Making, Color-Word Inhibition and Switching, Twenty Questions, Verbal Fluency, Design Fluency, Tower Test, Word Context, and Proverbs. The 16 main achievement scores are listed in Table 1. Full details of the tests and scores are available in the test manual (Delis et al., 2001).

**Table 1:** Factor Structure for the Three-Factor A Priori and Modified CHC Models.

CHC factor	D-KEFS main achievement score
Fluid reasoning (Gf)	Sorting condition 1: Free sorting confirmed sorts Sorting condition 2: Free sorting description score Sorting condition 2: Sort recognition description score Tower test: Total achievement score Tower test: Move accuracy ratio Word context test: Total consecutively correct Twenty questions test: Total weighted achievement score proverb Test total achievement score: Free inquiry
Processing speed (Gs)	Trail Making Test Condition 4: Letter-Number Switching Color-Word Inference Test Condition 3: Inhibition Color-Word Inference Test Condition 3: Inhibition/Switching
Retrieval fluency (Gr)	Verbal fluency test condition 1: Letter fluency total correct Verbal fluency test condition 2: Category fluency total correct Verbal fluency test condition 3: Category switching total correct Verbal fluency test condition 4: Category switching total switching accuracy Design fluency: Total correct design composite score

*Note.* CHC = Cattell–Horn–Carroll; D-KEFS = Delis–Kaplan Executive Function System.

In completing the analyses for the present study, a formatting error was noted in the D-KEFS standardization data table for the 8- to 19-year cohort and was subsequently confirmed by staff at Pearson Clinical Assessment (L. Whipple Drozdick, personal communication, October 27, 2021). This error involved the data rows for *Towers: Main Achievement Score* and *Towers: Move Accuracy Ratio* in Table 3.28. A corrected table was kindly provided by Pearson. As McFarland’s (2020) analysis was based on the published standardization data, apparently including the formatting error, we chose to run the replication models in this study using the original tables with the error first and then verify the results using the corrected tables. The same procedure was used for the CHC models. The reported results for the final CHC model in *Results* are based on the corrected tables.

## Procedure and Results

All models were initially estimated in the 20- to 49-year cohort with the number of observations specified at the minimum sample size (*N* = 361) in line with the recommendations of Marsh (1998). Sensitivity analyses revealed no significant change in model parameters or fit statistics for any model below, in any age cohort, when estimated with the maximum sample size for each indicator.

### McFarland Replication Models

The first objective was to replicate the preferred seven-factor model reported by McFarland (2020), namely the “7-Backwards-Correlations” model. As noted above, the method of model specification and estimation used by McFarland created doubts about the empirical accuracy and admissibility of this model but, notwithstanding these reservations, a conscientious attempt was made to replicate the model in two ways. As reported in McFarland (2020), this model included multiple loadings on each of the factors although many of the reported loadings may not have been significant because some loadings were small and significance was not reported. Based on the indicator-to-factor structure reported for this model (Table 4 in McFarland, 2020), the first method of replication involved assigning the largest factor loadings reported for each indicator in McFarland’s Table 4, together with the restricted pattern of factor correlations reported in his Table 5, to form a simple structure model to test using CFA. The second method instead sought a direct replication of the model, by nominating starting values of the factor loadings for every indicator on every factor as reported in McFarland’s Table 4. However, neither of these methods resulted in successful estimation of the 7-Backwards-Correlations model, with all CFA models failing to converge after 100,000 iterations. The same results were obtained using both the corrected and uncorrected data sets for the 8- to 19-year cohort. Such failure of convergence suggests that the proposed models were either mis-specified or not identified, or both.

The same procedure was followed to evaluate versions of the “7-Forward” and “7-Backward” models (Table 2 and Table 3, respectively, in McFarland, 2020). The first method involved assigning indicators to factors based on the largest factor loading reported for each model in McFarland’s corresponding tables. There were no factor correlations specified for the 7-Forward or 7-Backward model. In the second method, again starting values were nominated for factor loadings for each indicator in the corresponding tables. However, all of these models also failed to converge after 100,000 iterations when estimated with CFA. All analyses resulted in nonconvergence, whether the published or corrected correlation matrix was used (see above). As a consequence, it was concluded that McFarland’s (2020) reported models were not statistically replicable or clinically useful models of the D-KEFS scores.

**Table 2:** Global Fit Statistics for Each of the CFA Models in Each Age-Cohort.

Model	Chi squared				RMSEA				CFI				TLI			
	8–19	8–19 (corrected)	20–49	50–89	8–19	8–19 (corrected)	20–49	50–89	8–19	8–19 (corrected)	20–49	50–89	8–19	8–19 (corrected)	20–49	50–89
1-Factor General (g) a priori	2,121.68* *df*(104)	1,775.04* *df*(104)	1,229.78* *df*(104)	973.86* *df*(104)	.17 [.16–.17]	.15 [.15–.16]	.17 [.17–.18]	.16 [.15–.17]	.55	.59	.58	.66	.49	.53	.52	.61
1-Factor General (g) modified	459.36* *df*(94)	468.22* *df*(94)	314.87* *df*(94)	201.21* *df*(94)	.07 [.07–.08]	.08 [.07–.08]	.08 [.07–.09]	.06 [.05–.07]	.92	.91	.92	.95	.90	.88	.90	.95
3-Factor CHC a priori	1,081.54* *df*(101)	732.11* *df*(101)	558.43* *df*(101)	569.84* *df*(101)	.12 [.11–.12]	.09 [.08–.10]	.11 [.10–.12]	.12 [.11–.13]	.78	.85	.83	.82	.74	.82	.80	.78
3-Factor CHC modified	688.08* *df*(98)	338.58* *df*(98)	231.89* *df*(98)	183.35* *df*(98)	.09 [.09–.10]	.06 [.05–.07]	.06 [.05–.07]	.05 [.04–.06]	.87	.94	.95	.97	.84	.93	.94	.96
McFarland’s 7-factor backwards						No convergence in any sample										
McFarland’s 7-factor forwards						No convergence in any sample										
McFarland’s 7-factor backwards correlated						No convergence in any sample										

*Note.* 8–19 year cohort, *N* = 702; 8–19 year cohort (corrected), *N* = 702; 20–49 year cohort, *N* = 361; 50–89 year cohort, *N* = 326. CFA = confirmatory factor analysis; RMSEA = root mean square error of approximation; CFI = comparative fit index; TLI = Tucker–Lewis Index; CHC = Cattell–Horn–Carroll.

* *p* < .001.

**Table 3:** Standardized Factor Loadings and R^2^ Values for Three-Factor Modified CHC Model in Each Age Cohort

D-KEFS scores	Factor 1 (fluid reasoning)				Factor 2 (processing speed)				Factor 3 (retrieval fluency)				*R* ^2^			
	8–19	8–19 (corrected)	20–49	50–89	8–19	8–19 (corrected)	20–49	50–89	8–19	8–19 (corrected)	20–49	50–89	8–19	8–19 (corrected)	20–49	50–89
Sorting condition 1: Free sorting confirmed sorts	.728	.708	.699	.753									.503	.501	.488	.567
Sorting condition 2: Free sorting description score	.743	.719	.718	.757									.552	.518	.516	.573
Sorting condition 2: Sort recognition description score	.762	.716	.797	.739									.580	.513	.634	.547
Tower test: Total achievement score	–.098	.214	.341	.523									.010*	.046**	.117	.273
Tower test: Move accuracy ratio	.469	–.153	–.095	–.341									.220	.023*	.009*	.116
Word context test: Total consecutively correct	.446	.544	.571	.698									.199	.269	.326	.487
Twenty questions test: Total weighted achievement score	.369	.351	.378	.355									.136	.123	.143	.126
Trail making test condition 4: Letter-number switching					.536	.533	.682	.744					.287	.284	.465	.553
Color-word inference test condition 3: Inhibition					.607	.610	.511	.584					.369	.372	.261	.341
Color-word inference test condition 3: Inhibition/switching					.452	.457	.587	.543					.204	.208	.344	.295
Verbal fluency test condition 1: Letter fluency total correct									.717	.719	.704	.713	.514	.518	.496	.508
Verbal fluency test condition 2: Category fluency total correct									.753	.749	.772	.699	.567	.562	.595	.489
Verbal fluency test condition 3: Category switching total correct									.592	.591	.597	.496	.351	.350	.357	.246
Verbal fluency test condition 4: Category switching total switching accuracy									.433	.430	.518	.398	.187	.185	.268	.159
Design fluency: Total correct design composite score									.303	.306	.425	.498	.092	.094	.180	.248

*Note.* All loadings and *R*^2^ values are significant (*p* < .001) with the exception of the *Tower Test: Move Accuracy Ratio R*^2^ value in the 20- to 49-year-old sample (*p* = .410) and 18- to 19-year cohort (*p* = .069) marked with an *. The *R*^2^ value for *Tower Test: Total Achievement Score* in the 8- to 19-year cohort (corrected) was significant at *p* = .009, marked with a **. CHC = Cattell–Horn–Carroll; D-KEFS = Delis–Kaplan Executive Function System.

### CHC Models

A three-factor CHC model was specified a priori by the first and fifth authors using definitions for CHC constructs provided by Schneider and McGrew (2018) and the description of the 16 test scores in the D-KEFS manual (Delis et al., 2001), then verified by the second and third authors. The 16 scores were assigned to one of three factors a priori representing broad CHC abilities of fluid reasoning, processing speed, and retrieval fluency. The proposed three-factor structure for this CHC model is shown in Table 1.

**Figure 1.**
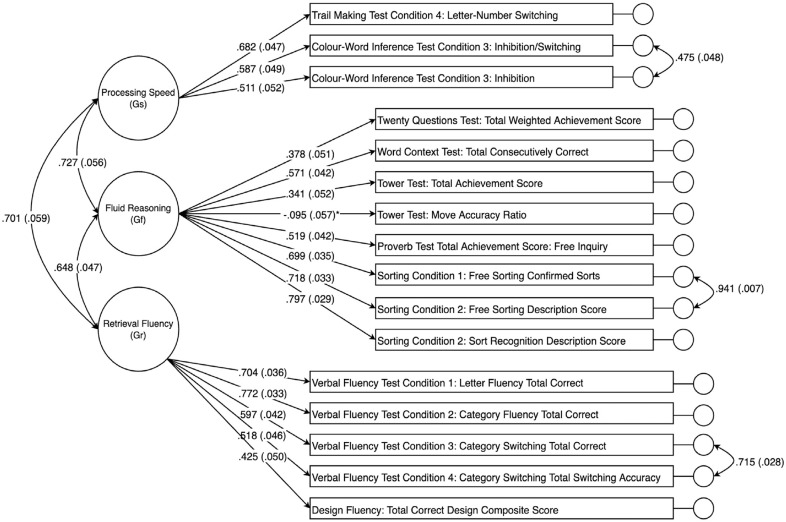
Path Diagram for the Three-Factor Modified Cattel–Horn–Carroll (CHC)-Guided Confirmatory Factor Analysis (CFA) Model in the 20- to 49-Year Cohort. *Note.* Including standardized factor loadings and standard errors in parentheses. All loadings were significant at *p* < .001 except Fluid Reasoning on *Tower Test: Move Accuracy Ratio* (*p = .099).

The a priori CHC model was estimated using CFA in the 20- to 49-year cohort first, chosen arbitrarily as the validation sample. Results indicated that the a priori model provided poor fit in terms of the RMSEA, CFI, and TLI values, which are shown in Table 2. As predicted, inspection of the modification indices showed potential for improvement in fit by inclusion of correlated residuals for many of the scores derived from the same or similar tests. As such, these correlated residuals were freed for estimation one at a time, in order of size of the modification index, until good-to-excellent global fit was achieved. The resulting post hoc model was then replicated in the 8- to 19-year cohort and the 50- to 89-year cohort. Although modification indices suggested that there were many possible correlated residuals that could be included to improve fit in each sample, only correlated residuals that replicated across each sample were included in the final post hoc model. These correlated residuals were between the following scores, in order of magnitude of modification index (a) *Sorting Condition 1: Free Sorting Confirmed Correct Sorts* with *Sorting Condition 1: Free Sorting Description Score*, (b) *Verbal Fluency Test Condition 3: Category Switching Total Correct Response* with *Verbal Fluency Test Condition 4: Category Switching Total Accuracy*, and (c) *Color-Word Inference Test Condition 3: Inhibition* with *Color-Word Inference Test Condition 4: Inhibition/Switching*. The path diagram for the final modified CHC model specified in the 20- to 49-year cohort is shown in Figure 1.

Global fit statistics for the a priori and modified three-factor CHC models are shown in Table 2 for each sample. Note that the modified model, which shows good to excellent fit in every sample, was modified post hoc in the 20- to 49-year cohort to iteratively include the three sets of correlated residual terms. Nevertheless, the resulting modified model with the three sets of correlated residuals was replicated as an a priori model in the 8- to 19-year and 50- to 89-year cohorts and provided significantly better fit than the original a priori model in each age group (all chi-square difference tests, *p* < .001).

A single-factor model representing general ability was also tested, which was specified by assigning all indicators to load onto a single factor. However, global fit statistics for this model revealed poor overall fit, also shown in Table 2. The addition of the same set of correlated residuals as incorporated into the three-factor model above did not lead to satisfactory fit (modified single-factor model in Table 2). Direct comparison of chi-square values and respective degrees of freedom between the single-factor model and three-factor model showed significant loss of fit for the single-factor model in each age-group cohort (*p* < .001 for all groups except 50–89 years, in which *p* = .002). Factor loadings and *R* ^2^ values for the preferred, modified three-factor CHC model with correlated residuals are shown in Table 3. The majority of these values were significant across all age cohorts, with a mixture of high and low values depending on the score. Again, the pattern of relative fit was preserved whether the published or corrected correlation matrix was used. All tables and the figure show results for the corrected data table.

### Bi-Factor Models

To be comprehensive, and despite our reservations regarding the conceptual and empirical application of bi-factor models of cognition, bi-factor model variants of both the a priori (without correlated residuals) and the modified (with correlated residuals) three-factor CHC models were also tested in each of the three age groups. In bi-factor models, all indicators are constrained to load onto a common general factor as well as their respective primary (group) factors and all factors are uncorrelated (orthogonal). However, all bi-factor models failed to converge after 100,000 iterations, suggesting these models were not well specified or statistically inadmissible and therefore not appropriate for the D-KEFS data.

We also tested the bi-factor measurement model reported by Karr et al. (2019). This bi-factor model included a general executive factor and three narrower (group) factors, referred to as inhibition, fluency, and shifting. In the study by Karr et al., nine D-KEFS scores were used to estimate the bi-factor model, which were calculated using the raw data and some transformed scores from the 20- to 49-year cohort in the D-KEFS standardization sample. The inhibition factor included three scores, including *Color-Word Inference Test Condition 3: Inhibition, Color-Word Inference Test Condition 4: Inhibition/Switching*, and *Tower Test: Total Achievement Score.* The fluency factor included *Verbal Fluency Test Condition 1: Letter Fluency—Total Correct, Condition 2: Category Fluency—Total Correct*, and *Design Fluency: Total Correct Designs*. The switching factor included *Trial Making Test Condition 4: Number-Letter Switching, Design Fluency: Switching—Total Correct, Sorting Test Condition 1: Free Sorting Confirmed Correct Sorts.* The *Design Fluency: Switching—Total Correct* score is not a main achievement score. Therefore, we were unable to perform a direct replication of the model using the D-KEFS standardization sample correlation matrices reported in the technical manual (Delis et al., 2001), which includes the 16 main achievement scores. However, we sought to replicate the preferred bi-factor model of Karr et al. (their Figure 2) in two ways. First, using the best available corresponding scores from the test manual which, as noted above, differ slightly because of differences in the scores available in the test manual versus the scores used by Karr et al. derived from the raw data. Second, we sought to estimate the bi-factor model (Karr et al., their Figure 2) using the correlation matrix reported by Karr et al. (their Table 2) which should faithfully reflect their data set. For both variants, the attempted replication failed to converge after 1 million iterations, with a variety of error messages reported including (a) negative residual variances, (b) not positive definitive covariance matrix, and (c) standard errors not estimated. Taken together, these results all suggest that the bi-factor model reported by Karr et al. is not admissible, and not well specified, despite our using essentially the same data set from which Karr and colleagues reporting their model.

### Measurement Invariance

Measurement invariance was examined separately for the 8 to 49 and the 50- to 89-year-old cohorts, respectively, compared to the 20- to 49-year cohort, for the modified three-factor CHC model. In line with previous analyses, the number of observations was specified at the minimum sample size for each cohort following the recommendations of Marsh (1998). Based on the procedure described by Widaman and Reise (1997), incremental equality constraints were applied to model parameters in four steps to examine the level of invariance across groups. Briefly, in Step 1, a baseline model was simultaneously estimated in the 20- to 49-year cohort and the comparison cohort to establish configural invariance. In Step 2, factor loadings were constrained to equality across groups to test weak metric invariance. In Step 3, factor loadings and intercepts were constrained to equality across groups to test strong metric invariance. Finally, in Step 4, factor loadings, intercepts, and residual variances were constrained to equality across groups to test strict metric invariance.

To evaluate invariance at each step of parameter constraint, the chi-square difference test (Δχ^2^), ΔCFI, and ΔMcDonald’s NCI (ΔMc) were calculated for the more restricted model compared to the preceding model. Different cut-off values for ΔCFI and ΔMc have been proposed. Here, we used cut-off values of ΔCFI = .01 and ΔMc = .02 as proposed by Cheung and Rensvold (2002).

The Δχ^2^, ΔCFI, ΔMc, and global fit statistics for each invariance step are displayed in Table 4 for comparison of the younger and the older cohorts against the 20- to 49-year cohort. As it has been suggested that TLI values are not useful to establish measurement invariance, these values were not used for the invariance analyses (Cheung & Rensvold, 2002; Meade et al., 2008). Applying a Bonferroni-type correction to the alpha error rate for the six comparisons of invariance steps in Table 4, a corrected significance threshold of *p* < .009 for Δχ^2^ (.05/6 = .009, rounding up) indicates that the hypothesis of strong metric invariance was retained for the 20 to 49 years versus 8 to 19 years cohort comparisons. In contrast, the assumption of strict metric invariance was not retained based on the Δχ^2^, ΔCFI, and ΔMc values. Examination of the modification indices for this strict invariance model suggested that the residual variances for *Verbal Fluency Test Condition 3* and *Verbal Fluency Test Condition 4* as well as the *Proverbs* score failed the test of equality, implying that for these items the unique variance, reliability, or both differed across the middle and younger cohort.

**Table 4:** Results of the Measurement Invariance Analyses for the 20- to 49-Year-Old Cohort Compared With the 8 to 19 and the 50- to 89-Year-Old Cohorts, Respectively

Model	χ^2^	*df*	Δχ^2^	N1	N2	RMSEA [90% CI]	CFI	ΔCFI	Mc	ΔMc
20–49 years old vs 8–19 years old				702	361					
Configural invariance	570.475	196		702	361	.060 [.054–.066]	.945		.8385	
Weak metric invariance	592.838	209	*p = .050*	702	361	.059 [.053–.064]	.943	–.002	.8348	–.0037
Strong metric invariance	619.707	222	*p = .013*	702	361	.058 [.053–.063]	.941	–.002	.8294	–.0054
Strict metric invariance	693.533	238	*p < .001*	702	361	.060 [.055–.065]	.933	–.008	.8071	–.0223
20–49 years old vs 50–89 years old				361	326					
Configural invariance	415.244	196		361	326	.057 [.049–.065]	.958		.8525	
Weak metric invariance	415.092	209	*p = 1.00*	361	326	.058 [.051–.065]	.954	–.004	.8607	.0082
Strong metric invariance	471.547	222	*p < .001*	361	326	.057 [.050–.064]	.952	–.002	.8339	–.0268
Strict metric invariance	505.001	238	*p = .006*	361	326	.057 [.050–.064]	.949	–.003	.8234	–.0105

*Note.* A Bonferroni-type correction to the alpha error rate for the six comparisons of invariance steps was applied, resulting in a corrected significance threshold of *p* < .009 for Δχ^2^ (.05/6 = .009, rounding up). RMSEA = root mean square error of approximation; CFI = comparative fit index.

Examination of the invariance steps for the comparison of the 20- to 49-year cohort versus 50- to 89-year cohort (Table 4) suggested that the hypotheses of strong and strict metric invariance could not be retained. Again, examination of the modification indices for the respective models suggested that the intercepts for *Verbal Fluency Test Condition 3* and *Verbal Fluency Test Condition 4* failed the test of invariance, indicating unequal difficulty for these items in the older cohort compared to the reference, middle cohort. Examination of the modification indices for the residual variances showed that the unique variance or reliability, or both, for the *Twenty Questions* score varied across cohorts.

## Discussion

The results of the present study support a three-factor cognitive ability model for the D-KEFS tests. As expected, each of the seven-factor models reported by McFarland (2020) failed to converge when replicated using the recommended approach to factor analysis (T. A. Brown, 2015; Strauss & Smith, 2009). Although the a priori three-factor CHC model was not found to be well fitting, the addition of correlated residuals based on large modification indices resulted in a well-fitting, theoretically coherent model that was replicable across all three age cohorts reported in the D-KEFS manual. Accordingly, this modified three-factor CHC model was selected as the best-fitting model for the D-KEFS standardization sample data. In line with many previous studies (for reviews, see Jewsbury et al., 2017), the results of the best-fitting model suggest that the D-KEFS measures three well-established constructs that have been described for many years under the CHC framework as general processing speed, fluid reasoning, and fluency (Flanagan & McDonough, 2018).

The current findings are partially consistent with previous factor analytic studies that have suggested a three-factor model for the D-KEFS based on switching, inhibiting, and updating constructs (Karr et al., 2019; Latzman & Markon, 2010). Jewsbury and colleagues (2016) previously demonstrated that the executive function constructs of switching, inhibiting, and updating are consistent with established cognitive abilities in the CHC model but are best described as general processing speed, narrow decision speed, and working memory, respectively. Latzman and Markon (2010) suggested that the D-KEFS factor structure may be described in terms of switching, inhibiting, and updating, but did not include a fluency construct, which was recently delineated in the CHC model and incorporates evidence from many years of clinical research (Jewsbury & Bowden, 2016; Schneider & McGrew, 2018). In contrast, Karr et al. (2019) supported a three-factor bi-factor model including switching, inhibiting, and fluency constructs, with fluency used as a proxy for updating based on some conceptual overlap between constructs. However, an attempted replication of this bi-factor model in the present study resulted in a lack of convergence after 1 million iterations, suggesting that bi-factor models are not admissible (statistically feasible) and may not be ideal for the D-KEFS data. This result may not be surprising since Karr et al. reported that only 65% of their bootstrapped samples led to convergence of their bi-factor model. In other words, the probability of their model converging in any single sample was not very different from 50/50 or chance, further suggesting that their model was not ideal. In contrast, the best fitting CHC model converged in each of the three samples examined.

The a priori three-factor model identified in the present study including the constructs of fluid reasoning, processing speed, and retrieval fluency provides support for the CHC model as a conceptual basis for the measurement model underlying the D-KEFS tests. The modified three-factor CHC model in the current study conforms to recommended guidelines for construct validation using CFA, derived from a theoretically guided, simple structure model that is well suited to clinical test score interpretation (T. A. Brown, 2015; Strauss & Smith, 2009).

The primary objective of the present study was to investigate the replicability of the seven-factor models proposed for the D-KEFS by McFarland (2020). McFarland criticized the factor analytic approach taken by Jewsbury and colleagues (2016), arguing that a simple structure factor model was insufficient to capture the nature of executive function as measured by the D-KEFS and that a complex model should be sought instead. However, each of these seven-factor models proposed by McFarland were unable to be estimated in the present study, likely due to model specification errors, despite being estimated using the same data sets as that used by McFarland. This failure to replicate McFarland’s models is likely due to the unconventional approach to factor analysis reported by McFarland, which involved unusual model selection criteria, idiosyncratic post hoc modifications, and other variations from recommended guidelines for conducting factor analysis (e.g., T. A. Brown, 2015; Kline, 2015; Widaman, 2012).

Turning to the clinical implication for use of the D-KEFS, the present findings also raise concerns regarding the recommended scoring procedure for the D-KEFS tests. Analysis of the a priori three-factor CHC model revealed large residual variances and low *R* ^2^ values for some of the indicators, suggesting that the common variance captured by some these test scores is low (Figure 1). However, the low model-attributable variance in scores is not likely to be a function of poor CHC model specification. Rather, the lower reliabilities of many of the D-KEFS scores (Delis et al., 2001) places a ceiling on convergent validity coefficients evident in the factor loadings (Figure 1). Notably, the *Tower: Move Accuracy Ratio Score* did not load significantly on the Fluid Reasoning factor, a low loading that may be a function of the small reliable variance component of this score, although no reliability estimate was reported for this score in the test manual. However, the loading of the *Tower: Total Achievement Score* approximated the reliability of this score that was reported in the manual as varying from α = .38 to α = .51 in different samples, reflecting poor reliability for this item. In addition, examination of modification indices gave no indication that the *Tower: Move Accuracy Ratio Score* should be assigned to a different factor. It should be noted that the problem of low reliability of executive tests is not limited to the DKEFS (Calamia et al., 2013).

Overall model fit improved when correlated residuals for scores from the same tests were freed for estimation, suggesting that residual variances may be representative of method or test-specific effects (Figure 1). For example, large correlated residuals were observed in all samples between the *Verbal Fluency Test Condition 3: Category Switching Total Correct* and *Verbal Fluency Test Condition 4: Category Switching Total Switching Accuracy* (*r* = .64 to *r* = .72). These scores represent different ways of measuring performance on the same test trial, with both scores loading onto the retrieval fluency factor in the final model, suggesting the scores measure the same broad cognitive ability. Three correlated residuals assumed to represent method effects were included in final modified three-factor CHC model. Method effects such as those described in the previous example can be mistaken for discrete cognitive constructs if factor analysis is not applied carefully. For example, in the bi-factor model reported by Karr et al. (2019), residual variance was captured by an additional bi-factor, representative of common executive function, which loaded onto each indicator in addition to the three primary factors. While method effects can be accounted for with sensible inclusions of correlated residuals in a simple structure model, bi-factor models are not readily interpretable because in a bi-factor model each item is presumed to represent two uncorrelated abilities (Greene et al., 2019). The failure of the bi-factor model reported by Karr et al. (2019) to converge in the current study provides further evidence that bi-factor models may not suitable for the D-KEFS data.

As highlighted by McFarland (2020), the D-KEFS tests were not designed to test discrete latent variables. The D-KEFS tests were selected or modified for inclusion in the battery based on clinical expertise about tests that are sensitive to the type of executive impairment commonly observed in individuals with brain disorders (Lezak et al., 2012), despite concerns regarding the reliability or validity of some of the tests (Crawford et al., 2008; Schmidt, 2003). Furthermore, the 16 main achievement scores described in the technical manual are not intended to measure distinct executive function constructs, but are assumed to each be influenced by a range of executive processes (Delis et al., 2001, 2004). This point is of particular interest, because without an underlying measurement model it is unclear what the test scores measure. In addition, emerging evidence suggests that executive tests can, in fact, be incorporated into established cognitive ability models that build on many years of construct validity research and augment the historic constructs identified from general cognitive ability batteries (Connolly et al., 2020; Flanagan & McDonough, 2018; Jewsbury & Bowden, 2016; Jewsbury et al., 2016; Schneider & McGrew, 2018). The results of the current study suggest that a relatively small set of well-researched cognitive abilities may account for most of the reliable variance in D-KEFS subtest scores and that there is considerable redundancy in the multiple scoring approach.

Moreover, examination of measurement invariance suggested that the three-factor CHC model generalized with numerical precision across the three age cohorts (Table 4). Although there were a small number of specific item parameters that failed the tests of invariance, as described above, weak metric (factor-loading) invariance was demonstrated across the three cohorts (Table 4). Such a finding, in addition to demonstrating that the latent factor model is robust across the adjacent age cohorts, shows that convergent and discriminant validity evidence generalizes across the cohorts. In other words, convergent and discriminant validity correlations can be interpreted as indicating similar construct validity evidence in the three cohorts. The demonstration of strong metric invariance across younger and middle age cohorts, and middle versus older age cohorts, with the exception of evidence of unequal item difficulty (or differential item function) for the *Verbal Fluency Test Condition 3* and *Verbal Fluency Test Condition 4* scores in the latter comparison, indicates that mean scores comparisons can be interpreted in uncomplicated fashion across the three cohorts. The present findings suggest that administering all nine D-KEFS tests may be unnecessary and may impose undue expenditure of time and effort for patients. Although the test manual encourages clinicians to administer a selection of D-KEFS tests based on the needs of individual patients rather than the full battery, this approach places an overreliance on clinical expertise and subjective judgment. The results of this study suggest that scoring for the D-KEFS can be rationalized using three latent variables, namely, the CHC broad abilities fluid reasoning, processing speed, and retrieval fluency. The most cost-effective approach may be to administer the single, most reliable and best indicator of each latent variable. Clinicians should focus on retest reliability rather than internal consistency since the latter is no longer regarded as a useful guide to the clinical or applied concept of reliability (Sijtsma, 2008). Although the data set presented here cannot estimate the retest reliability of any indicators, Figure 1 nevertheless shows that the strongest and best indicator of processing speed is Trails, of fluid reasoning is Sorting, and of fluency is Verbal Fluency. Assimilating the D-KEFS with CHC theory allows clinicians to make rational choices about tests that measure CHC constructs based on the available criterion validity evidence. Clinicians can then choose the single best indicator or a combination of indicators to obtain a reliable and hence precise estimate of the construct of interest (Nunnally & Bernstein, 1994).

Our findings imply that executive function is not a single construct, nor is it a construct separate from the well-defined CHC cognitive abilities, as has been previously considered (e.g., Salthouse, 2005). Instead, our results support the findings of Jewsbury et al. (2016), who advocated for the integration of executive function constructs with CHC theory. Importantly, and as a direct demonstration of the strength of the CHC model, all conceptual predictions of D-KEFS test score to construct (factor) assignment, made a priori on the basis of CHC definitions, were shown to be correct. No test score needed to be re-assigned to other factors, as all factor assignment predictions were supported by the data in all three samples. Given that executive function is commonly assessed alongside other cognitive abilities in standard neuropsychological assessments, the strength of having the CHC model as a single cognitive ability model to interpret performance for all tests is clear. Using the well-established and empirically validated CHC model as a basis for test scoring and interpretation would therefore provide a coherent framework for clinicians to interpret and compare individual performance across many aspects of cognitive ability. The results may also be interpreted as compatible with precursors or prior variants of the CHC model, such as Cattell and Horn’s *Gf-Gc* model (Cattell, 1957) and Carroll’s Three Stratum model (Carroll, 1993), which are not mutually exclusive with the current model. However, a comparative evaluation of the CHC model and historical precursors was not feasible in the present study in view of the limited number of factors available to be modeled. Instead, the current study aimed to test the compatibility of CHC constructs with the D-KEFS tests while evaluating the McFarland (2020) analysis.

It can be noted, however, that the CHC model was compiled from conceptual integration of previous factor analysis studies and is strikingly similar to the conceptual model of cognitive abilities reported in *Diagnostic and Statistical Manual of Mental Disorders* (5th ed.; *DSM-5*; American Psychiatric Association, 2013), with the conspicuous exception of the conflated construct of executive function described in *DSM-5*, and the as yet ill-defined concept of social cognition reported in *DSM-5* (see Frith & Blakemore, 2006; Vallat-Azouvi et al., 2019). There remain many opportunities for confirmatory evaluation of the detailed predictions of the CHC model (McGrew, 2009). Nevertheless, the use of the CHC model to predict factor assignment and construct validity, across a wide array of cognitive test batteries and populations, has proven remarkably successful (Agelink van Rentergem et al., 2020; Caemmerer et al., 2020; Goette, 2020; Jewsbury et al., 2017).

This study was limited by the use of the correlation and covariance matrices specified to two decimal places as provided in the D-KEFS standardization data. Analyses based on correlation matrices rounded to two decimals places may lead to imprecise estimates of covariance structures. It is recommended that three decimal places be used for increased precision of estimates. However, as this is an inherent limitation of the D-KEFS standardization data set, the present findings are subject to the same limitation as all previous findings that were based on the same data set.

Demonstrating the replicability of empirical findings is a core metascientific principle and necessary to demonstrate that the findings are credible rather than an artifact of a specific study. To our knowledge, this is the first validation study of the D-KEFS standardization sample using the covariance structure, which retains the natural dimensionality of the test scores (T. A. Brown, 2015). Using the same data as previous studies, we tested theoretically guided models and explored alterative solutions to previously published models which sought to reject the generality of the CHC predictions (McFarland, 2020). McFarland’s (2020) seven-factor models were not replicated, and this failure of replication underlines the importance of adopting recommended methods of factor analysis. The present study demonstrates that CHC theory appears to extend to the D-KEFS tests, supporting previous findings that executive function abilities can be integrated into CHC theory.
